# Soldering in Dentistry: An Updated Technical Review

**DOI:** 10.3390/jcm13030809

**Published:** 2024-01-30

**Authors:** Enzo Cumbo, Giuseppe Gallina, Pietro Messina, Giuseppa Bilello, Mohmed Isaqali Karobari, Giuseppe Alessandro Scardina

**Affiliations:** 1 Department of Precision Medicine in Medical, Surgical and Critical Care (Me.Pre.C.C.), University of Palermo, 90133 Palermo, Italy; enzo.cumbo@unipa.it (E.C.); giuseppe.gallina@unipa.it (G.G.); pietro.messina01@unipa.it (P.M.); giuseppa.bilello@unipa.it (G.B.); 2 Dental Research Unit, Center for Global Health Research, Saveetha Medical College and Hospital, Saveetha Institute of Medical and Technical Sciences, Chennai 600077, Tamil Nadu, India; dr.isaq@gmail.com

**Keywords:** gas welding, electric welding, laser welding dentistry, prosthodontics, orthodontics

## Abstract

Introduction: The need to permanently join two or more pieces of metal using heat is a frequent condition in various fields of medicine such as dentistry. Welding, brazing and soldering are permanent joining techniques between different metals that require in-depth background knowledge in order to obtain predictable results. Aim: This review examines the different methods of joining metals using heat and their fields of application. Discussion: It is possible to create permanent metal joints in various phases of the creation of final products that will be used on the patient. In several cases, welds are also made directly by the manufacturer during industrial processing. In dentistry, dental laboratories perform complex welds mainly on dental prostheses and orthodontic appliances during the production process. It is also possible to obtain intraoral welding carried out by the clinician inside the patient’s oral cavity. Welding can be carried out using combustible gases, electric current, infrared light and laser light through different technical procedures which must be chosen according to the specific needs and the metals to be joined. Conclusions: It is useful for the dentist and dental technician to know the different welding methods, including those carried out in the factory by the manufacturer, to better understand the physical properties and mechanical resistance of the components marketed for the construction of prostheses and orthodontic appliances. The enormous variety of conditions in which those who practice welding can find themselves therefore presupposes in-depth knowledge in this field in order to apply the most suitable technique.

## 1. Introduction

Welding, soldering and brazing are all techniques for joining two or more pieces of metal together using heat. They are used in various fields, including bioengineering and medicine, to permanently join metal parts [1]. Welding is a fundamental technical process in the construction of various metal objects whose components must be mechanically resistant and durable.

To create a definitive joint, a device is needed that produces heat and pressure to transfer to the metal parts to be joined together.

The main difference between welding and soldering is fusion. In soldering, the operator heats the metal parts to be joined but never softens them. In welding, however, the metal parts are melted by the heat. Brazing consists of heating and melting a filler alloy. Once the filler solidifies, the metal pieces are joined together [2].

Welding. The purpose of welding is to create a very strong bond between two pieces of metal that can withstand all kinds of stresses and strains. The two or more metals must be similar, and they are heated to a very high temperature so that the two parts merge and join. This is a process of joining pieces of metal using temperatures higher than the melting temperature of the metals to be joined, causing them to melt.

If filler material is used, it is also melted, favoring the filling of any spaces between the parts to be joined and improving the mechanical characteristics of the joint. When the molten metals solidify, a strong and lasting connection is created [3]. 

There are two different types of welding: autogenous and heterogeneous [4]. Autogenous welding is a welding process in which the welding material is obtained from the same metal as the work piece being welded. In other words, the weld material and the material of the work piece being welded are the same. Autogenous welding is often used to weld materials such as iron and steel. Heterogeneous welding, on the other hand, is a welding process in which the weld material is different from the material of the work-piece being welded. For example, an alloy of dissimilar metals might be used to weld two pieces of steel together. This type of welding is often used to weld materials that are difficult to join through the autogenous process, or to obtain specific characteristics at the weld point. Depending on the type of welding, the filler material, which can come in different forms such as a wire, metal rod or even a coated electrode, is then used to bridge the space between the pieces and strengthen the joint [5].

Soldering. Soldering is used mostly in electronic devices, where it allows the components to connect with each other electrically. Aesthetically, it may look similar to welding, but the filler material used in this technique is quite soft and usually comes in rods and coils.

Brazing. It should be noted that, according to the relationship between the melting temperature of the metals involved and the temperatures reached by the technique used, it is necessary to differentiate actual welding from brazing. Brazing is a process of joining two or more pieces of metal using a filler material called a brazing alloy which melts at a lower temperature than the metals being joined. Brazing is often used to join metals that are difficult to weld or cannot be joined by the standard welding process due to their chemical or mechanical properties (Table 1) [6,7]. 

## 2. Welding Application Fields

Different types of metal joints obtained through heat are used in medicine and therefore in dentistry. Some of them are performed by the manufacturing factories, for example, during the construction of prefabricated prosthetic parts. However, sometimes, the processes of making complex prostheses or orthodontic appliances requires welding to be carried out in dental laboratories or dental offices in order to customize and optimize the final product [8].

In some cases, it is even possible to proceed with a particular welding called “intraoral” to join together different prosthetic components inside the oral cavity. On some occasions, welds make it possible to correct errors made during the construction of dental products, such as prostheses, by separating the product that has manufacturing errors into several parts and rejoining the pieces in the correct position. In other clinical conditions, it is preferred, for example, to construct the metal part of a prosthesis that is divided into several components by joining together through welding. This method is preferable because the creation of a single large prosthetic metal component may have poor accuracy and/or deformations due to thermal stress during melting and casting of the metal [9].

If we exclude the silver solders used to join wires in orthodontics and the spot welding of orthodontic bands, most of the applications of the solder are in the field of the prosthesis.

Dentists and dental technicians are quite familiar with the processes of union and combining individual prosthetic components to be welded together to obtain the final product. However, in general dental practice, most clinicians consider the use of welding to be an emergency condition rather than an elective procedure. However, for expert technicians and specialized restorative dentists, welding is a fine art and an indispensable tool to improve precision in the realization of some manufactured goods such as very extensive fixed prostheses where the risk of having imperfections is very high.

It is useful for dentists and the dental technicians to know the different welding methods, even those carried out in the factory by the manufacturer, in order to better understand the physical and mechanical resistance properties of the components marketed for the construction of prostheses and orthodontic appliances.

## 3. Dental Alloys, Welds and Filler Metals

The metals used in dentistry are many, and their compositions are fundamental both to their physical and mechanical properties and to their workability, including their ability to be welded through the application of heat [10]. Indeed, the composition of metal alloys always determines their mechanical properties, melting range and oxidation potential [11]. Oxide formation affects the “wettability” and thus the ease of welding [12]. Commonly used alloys include ADA-rated yellow gold casting alloys, low-gold-content variants and alloys capable of bonding to porcelain. The alloys selected for intra-oral use must be non-toxic and resistant to oxidation and corrosion. This is the reason why the noble ones are preferred [13]. Alternatively, to reduce costs, so-called non-noble alloys can be used, which include cobalt and/or nickel [14,15,16]. Other alloys, widely used today in dentistry, consist of titanium and are used in implant prostheses [17]. Titanium combined with nickel is instead widely used in orthodontics.

Dental welding derives from jewelry manufacturing techniques, with the important difference that, in dentistry, precision, strength and corrosion resistance are very critical, while the aesthetic appearance is often less important because the area in which the joining it is performed is usually not visible [18,19].

Soldering plays an important role in dentistry, as evidenced by the large selection of solders and fluxes that are currently available from alloy suppliers. The filler metal is an alloy which, once melted, flows and wets the parts to be joined, then solidifies, forming the weld joint. The compositions of welding alloys are often different from those of the master alloys and are often eutectic. In fact, they are a mixture of substances with a resulting melting point that is lower than the single substances that compose them.

All solder materials should have specific characteristics [20], two of which are of fundamental importance: flowability and strength. Flowability is closely linked to the fact that alloys are eutectic. In fact, the higher the “delta temperature” relative to the melting point with the master alloy, the greater the flowability and the ease of carrying out the procedure. The filler alloy should freely flow and wet the metals, which will be joined through its penetration into the small cracks through capillary action at temperatures ranging from 50 °C to 100 °C below the solidus temperature of the latter. Generally, a weld with a narrow melting range has superior flow characteristics [21,22]. On the other hand, the strength should be similar to that of the master alloy. However, solder alloys are, in fact, modified versions of their master alloys, and as such, they are mechanically less resistant. Fortunately, joint strength is improved by heat, hardening and the phenomenon known as triaxiality.

Filler materials can also be divided into two main groups: soft and hard. Pb–Sn alloys in various composition ranges are an example of soft welding. They have a melting point of approximately 260 °C [500 °F] and are used to join lead, copper or brass. They have good processing and mechanical properties, but they cannot be used in dentistry due to their lead contents and poor corrosion resistance. Filler materials for dental use are, in fact, quite hard, having a much higher melting temperature and being mechanically very resistant [23]. This type of filler material includes those based on gold, those for pre- and post-ceramics and those based on silver. Silver is more subject to tarnishing and corrosion when used in the oral cavity. For this reason, it is not used for prosthetic applications, and it is mainly used in welding on orthodontic appliances. These alloys can also be used for joining stainless steel or other base metal alloys. More precisely, silver gas solders have a much higher strength and hardness than pure silver. They bond well with almost all metals including stainless steel. Some silver rods are supplied with a coating of deoxidizing powder, although in most cases, it is preferable to sprinkle the surfaces with borax powder which, in the flame, melts and chemically deoxidizes the metals. This is also because coated rods are generally more expensive and delicate.

Other filler materials used to join gold alloys are composed of gold, silver and copper, with small additions of other metals such as zinc and tin [24]. The melting range can be narrowed by lowering the gold content and increasing the copper content. Zinc and tin lower the melting point, and gold gives resistance to corrosion. Even if the exact minimum indispensable quantity of this noble metal is still the subject of discussion, it can be concluded that the percentage varies from 58% to 61% [25]. Depending on the metal and welding needs, one welding technique may be more suitable than another.

## 4. Gas Welds

**Figure 1:**
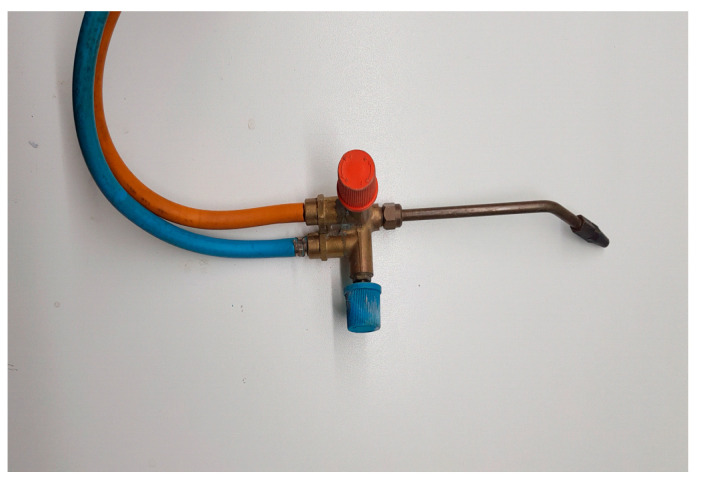
Gas welding torch.

Gas welding, also commonly known as torch welding, is a form of “strong” welding because the filler material is very strong and mechanically resistant (Figure 1) [26]. These metals generally have a melting temperature between 800 and 1000 °C and are in the form of rods with a small diameter of approximately 2 mm and a length of approximately 300 mm long. Additionally, it is necessary to use flames with a high calorific value.

Gas torches have, in the best models, a piezoelectric ignition system. Each click corresponds to a strong spark that strikes in front of the burner nozzle, and the flame is immediately ready. In addition to the gas torch, it is necessary to have a connection pipe comprised of gas-resistant rubber that is more than 2 m long and equipped with suitable connections [27].

The delivery devices intended for use with gas cylinders in welding are very accurate from the point of view of safety. In addition to the non-return valves, which prevent the occurrence of flashbacks, there are also pressure regulators, one for each cylinder, with a double pressure gauge, allowing operators to set the correct preconditions to manage the final calibration of the flame using the knobs at the top of the torch. Gas torches also have a very simplified fuel system. To carburize means to saturate a gaseous atmosphere, i.e., the air, with hydrocarbons. This saturation is adjusted so that the flame forms a bluish dart in its center, which has the maximum calorific value. Carburation usually takes place only by increasing or decreasing the gas flow, while the air enters through fixed nozzles that are arranged in the construction of the torch.

A liquified gas can reach 1800 °C in the dart, but due to inevitable dispersions, the useful temperature is reduced to approximately 1300 °C. There are many liquified combustible gases, although in most cases, butane and propane are used [28]. Propane is clearly superior to butane in terms of efficiency: it gasifies more easily, even when the ambient temperature is low. However, butane, on the other hand, is less expensive. Both these liquified gases have numerous advantages including easy portability and excellent conservation due to the cylinders being built in accordance with high safety standards [29].

On the market, there are a large variety of non-refillable cylinders with gas mixtures for welding. These often contain variable percentages of propane and butane. When it is necessary to reach higher temperatures, other components such as propyl and acetone are added, allowing the dart to burn up to 3000 °C. Other available gases that can be used, even when mixed with oxygen, include LPG and methane.

Another strategic gas that could be used as an alternative to propane and butane is oxyacetylene. This method involves oxyacetylene welding, where temperatures close to 3000 °C are involved [30]. Although acetylene is a gas that needs to be handled with extreme care, the cylinders have high safety standards, with the upper collar protected so that no blow, fall or other accident can damage the taps. The flame is produced at the end of a torch in which acetylene and oxygen combine in optimal ratios [52% acetylene and 48% oxygen] in order to produce a neutral flame. Acetylene is widely used due to its high flame temperature and high thermal content, as well as the low reactivity of the flame with the base and filler metal and ease of flame regulation. This method is convenient for small thicknesses, and it has the advantages of a portability of the equipment, low cost and the possibility of welding in all positions. 

The oxyacetylene dart has a length that varies according to the torch used. The temperature is higher closer to the torch nozzle. Depending on the color and shape of the flame, different characteristics can be achieved. A large and very bright yellow flame is oxidizing and too rich in oxygen, while a narrow, blue flame is reducing and rich in acetylene. To obtain a good weld, mixing must take place in the correct ratios [31]. The formation of the flame is obtained at the expense of both the acetylene and oxygen cylinders. Additionally, the oxygen present in the air contributes to the combustion. For the complete combustion of one liter of acetylene, 2.5 L of oxygen is required. Of this total amount, on average, 1.1 L is supplied by the cylinder and 1.4 L is supplied by the surrounding air. This concept shows how important it is to carry out gas welding in the presence of good ventilation.

After lighting the torch, the carburetion should be checked before swinging the flame to preheat the piece to be welded. A small amount of deoxidizing powder should be deposited on the point to be welded, then the flame is passed over it again, making it melt and boil. When the bubbles have almost completely disappeared, the first drops of metal can be dripped over the joint.

Sometimes, in order to weld correctly, it is advisable to perform spot welding. This involves joining two pieces together by dropping a drop of filler material at the two ends of the section to be welded, temporarily uniting the two parts. Once this result has been obtained, to avoid deformations caused by the heat, it is advisable to continue spot welding by dropping a few drops of filler material into intermediate areas.

**Figure 2:**
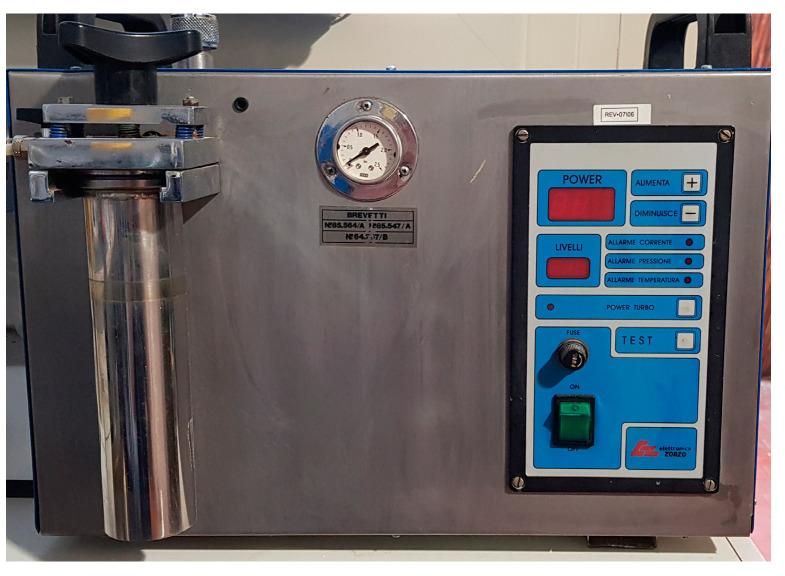
Hydrogen welder.

Among the gases used for welding, we must include hydrogen, which is abundantly present in water. Special welders, due to electrolysis, can be extracted and used to produce open flames (Figure 2) [32].

These hydrogen generators only using only water and electricity, producing hydrogen without storage and minimizing the risk of dangerous explosions. The mixture of hydrogen and oxygen present in the air produces a neutral flame which develops at a temperature of approximately 3650 °C. This can be used to weld and braze all non-ferrous materials in extremely short times compared to other welding systems [33]. The gas exiting the torch is perfectly mixed and does not require any adjustments by the operator. In this way, it is also simple to standardize the welding processes, also reducing oxidation phenomena. In addition, the torch is ergonomic and light, with a weight that is approximately 50% less than traditional torches. With hydrogen welders that use water, it is therefore possible to eliminate traditional cylinders and their risk of bursting and reduce expenses, as the purchase costs of other gases are eliminated. Moreover, the flame is ecological, because water vapor is generated from the combustion of hydrogen and oxygen, without producing CO_2_ emissions that are harmful to the environment and the operator. The internal pressure in the cylinder due to the temporary accumulation of gas in the generator is a maximum of 0.5 bar [34]. Another advantage is represented by the fact that the flame produced by hydrogen does not force the dental technician to wear dark protective goggles, which limit visibility. The flame is neutral and highly concentrated, allowing heating of only the affected part of the product without overheating a large surface. Thanks to the high temperature of the flame, the alloy penetrates deeper than traditional systems. This allows for more robust joints with less alloy waste [35,36].

## 5. Electric Arc Welding

This type of welding involves an electric current that passes through an ionized gas. The arc strikes between the electrodes (anode and cathode) when a temperature that allows the emission of electrons is reached, and it is automatically maintained in the presence of a potential difference. It is therefore necessary to obtain certain conditions of temperature, voltage, current intensity and ionization of the gas between the electrodes. Usually, the average temperature inside the arc is approximately 3800 °C.

Different welding methods have been developed based on the electric arc:

- *Cored wire* (flux-cored arc welding): In this method, the electric arc strikes between a metal electrode with a continuous power supply and the base material [37]. The electrode contains a flux, i.e., a chemical cleaning agent which prevents oxidation. In fact, the flux itself is relied upon to generate the necessary protection from the atmosphere, producing both gaseous protection and liquid slag protecting the weld. This process can occur with or without a protective gas.

The main advantage of this process is that it generally produces welds with excellent mechanical properties. Moreover, there is always a good penetration into the base metal with a low production of porosity [38]. This method is widely used on an industrial level because it is not necessary to continuously change the electrode. Moreover, it can be easily robotized, allowing for rapid production times.

- *MIG and MAG welding*. Flux core welding is similar to MIG/MAG welding. In fact, both of these methods use a spool of wire to provide the filler metal to the weld, but the primary difference is in the type of wire [39]. MIG welding uses a solid wire, whereas flux core welding wire is tubular, with the flux contained inside the tube. These acronyms, MIG and MAG, derive from the names “metal inert gas” and “metal active gas.” The MIG or MAG welding process, also known as continuous wire welding, is also similar to TIG. However, it differs in that it has a fusible electrode in the form of a wire, which also forms the filler metal. The difference between MIG and MAG essentially depends on the type of gas used. MIG welding uses inert shielding gases, while MAG welding uses active shielding gases. Inert gases do not take part in the reaction and do not change the result, unlike active gases. In the case of MIG welding, the protective gases are argon and helium. For MAG welding, on the other hand, protective oxidizing gases are used; often mixtures of argon and carbon dioxide [40].

The choice between MIG and MAG Is dictated by the type of metal to be welded. MAG welding, for example, is more suitable for the welding of carbon steels for two reasons: it has a greater penetration power and it makes the positioning of the electric more stable.

The transfer of the filler metal to the base metal can take place in various ways depending on the voltage of the arc and the current flowing through it. The “short arc mode” occurs for low arc voltages: the transfer takes place with the formation of large drops that stretch towards the base metal, creating a short circuit and momentarily extinguishing the arc. This phenomenon repeats itself between 20 and 200 times per second. The solidification of the weld bead is rapid; therefore, this mode is suitable for welding in any position and for thin materials.

The “spray arc mode” occurs with higher arc voltages and high currents. The transfer occurs in the form of a high number of drops with small dimensions which pass through the arc without extinguishing it. The fluidity of the weld pool is high, and the solidification of the weld bead is slow. For these reasons, it is only suitable for flat welding [41].

MIG/MAG welding has several advantages. Firstly, it is cheaper than other types of welding. Furthermore, the presence of the shielding gas guarantees that the process takes place without interference from any slag or oxygen. Finally, the continuous wire process makes it a flexible welding method in terms of use because it allows the user to weld any metal and it is suitable for high production rates. This is because it is not necessary to replace the electrode. MAG welding is suitable both for industrial processes for the production of preassembled dental components [by welding] in the factory and for manual welding in the dental laboratory.

**Figure 3:**
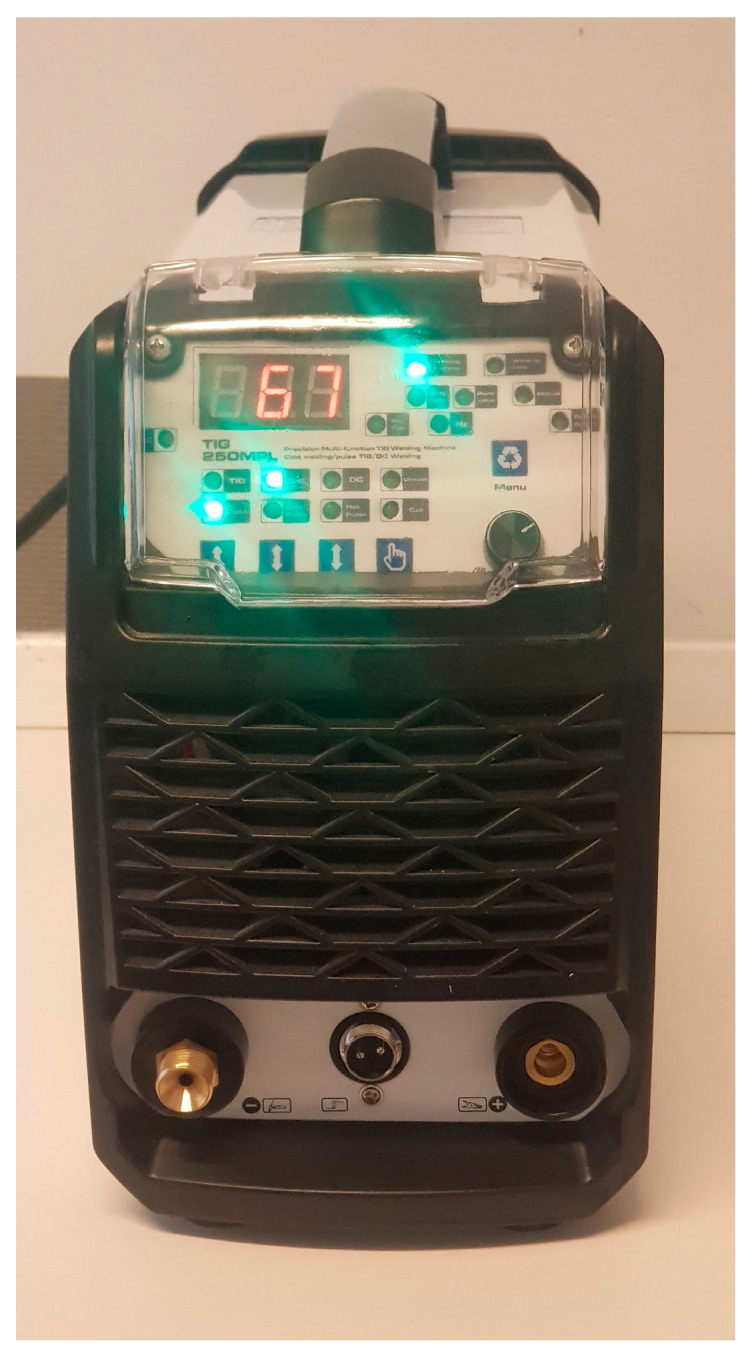
TIG welder.

- *Shielded Metal Arc Welding:* This process, also called manual metal arc (MMA), is a welding process based on a consumable electrode that is also covered by a flux [42]. An electric current, alternating or direct, is used to generate the arc. During execution, the coating of the electrode disintegrates, giving off vapors which serve as a shielding gas and providing a layer of slag. These phenomena both serve to protect the welding area from air contamination. The electrode consists of a core metallic wire covered with silicate binders and other material that may include fluorides, carbonates, oxides, metal alloys and cellulose. The cover is extruded over the wire and then dried in an oven. Although this welding method is one of the most widespread in the world, it is rarely used in the dental field, even in industrial production.
- *Submerged Arc Welding:* This method requires a solid or tubular electrode (flux cored) with a continuous power supply. The weld pool and arc zone are protected from atmospheric contamination by being immersed in a blanket of fusible granular flow made up of calcium, manganese or silicon oxide. When molten, the flux becomes conductive and provides a path for current between the electrode and the workpiece [43]. This thick layer of flux completely coats the molten metal, preventing splashes and sparks. It also suppresses the intense ultraviolet radiation and fumes that are part of SMAW. Welding can be carried out only in the flat position. This welding method is efficient but has no applications in dentistry.
- *TIG welding* (Gas tungsten arc welding): TIG welding is an electric arc welding process in an inert atmosphere (Figure 3). The arc is produced by the shielding gas (argon or helium) that comes out of the gun which also carries the electrode. The arc is ignited by a pilot spark, which, by causing ionization of the protective gas, makes it conductive. The electrode is made of tungsten, and given its high melting temperature, it does not melt. This process can take place with or without a filler metal [44].

TIG allows the technician to easily weld in all positions with excellent weld puddle control. This method is widely used in all quality welding [45,46,47]. The tungsten electrode is placed in proximity to but without physical contact with the structures to be welded. It triggers an electric arc capable of melting the metals at a given point. In particular, the calibration of TIG machines foresees currents between 50 and more than 100 amperes protracted for seconds, usually with negative polarity to the tungsten electrode and positive to the piece to be welded. This type of setting concentrates approximately two-thirds of the welding heat on the structure. The remaining third is concentrated on the tungsten. The increased heat input to the weld results in deep penetration [48].

The entire welding process takes place in a protected atmosphere. This prevents any tendency for oxidation, which normally occurs following an increase in temperature at the junction point.

The flow of protective gas must be kept within a range of between 4 and 10 L per minute so that the whole area affected by the welding process is completely invested by the jet and therefore continuously protected [49,50].

Among the possible adjustments, all TIG welders allow the argon gas output to be brought forward with respect to the electric arc ignition (pre-flow time) in order to ensure that the dome of inert atmosphere is well-formed before the moment of welding. For the same principle, it is possible to set how long the gas flow must continue after the arc has gone out (post-flow time) so that oxidation phenomena do not occur even when the welded pieces are cooling.

Usually, a 1.6 mm diameter electrode of the WL20 BLUE type with 2% lanthanum (LaO2: 1.8–2.2%) is inserted into the handpiece of the welding machine. This offers excellent ignition performance and durability over time. It maintains its clean shape and unaltered tip, and it does not contain thorium; therefore, it is not harmful to health or the environment [51]. It is also necessary to sharpen the tip of the tungsten electrode lengthwise, giving it the shape of a pointed cone. The sharp tip of the electrode provides a constant and concentrated arc in the workpiece. The sharpening angle and length affect the width of the arc and the depth of penetration.

## 6. Infrared Welding

Infrared welding is a non-contact welding technique used between the pieces to be welded in which an infrared emitter melts the areas to be welded. It is used to join metals when clean and precision welds are required [52]. Infrared rays are electromagnetic radiations that have a wavelength between 700 nm and 1 mm. Not all infrared rays hitting an object are absorbed, as a part is always rejected. It is therefore necessary to study the chemical–physical characteristics of the material to be welded and, based on these characteristics, to design an emitter that guarantees the greatest possible absorption of radiation.

**Figure 4:**
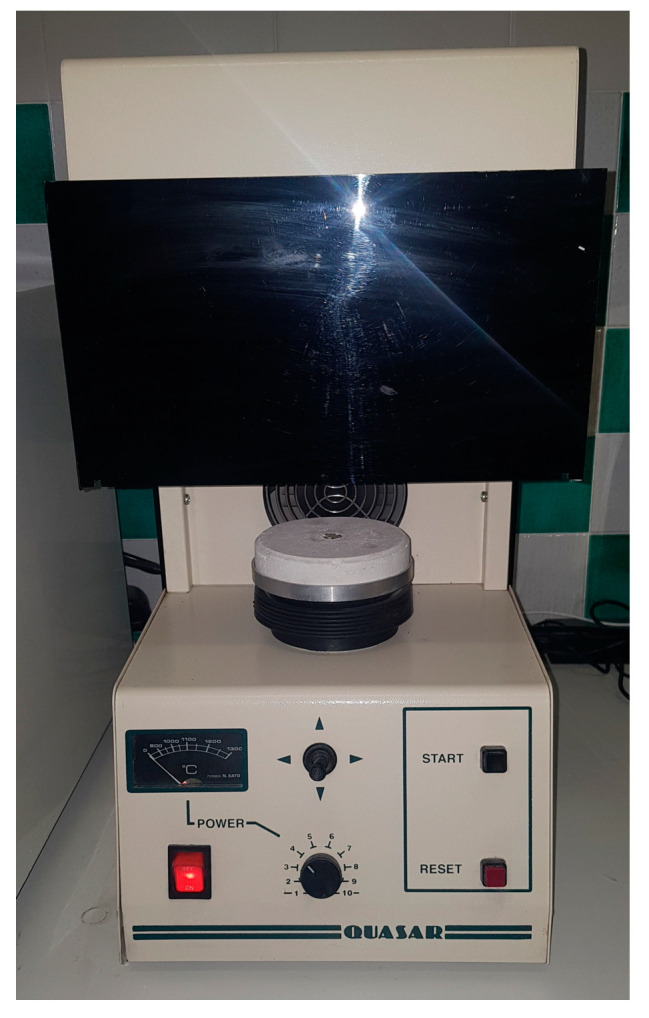
Infrared welder.

The production of thermal energy takes place through an infrared optical system that is capable of generating temperatures of approximately 1350 °C which, thanks to a system of parabolic mirrors, concentrates all the thermal energy (with an optimal focus) on a small surface of approximately one centimeter in diameter (Figure 4).

Normally, a power control system, which determines the temperature and heat that are gradually generated, heats the elements to be welded without altering the crystalline structure of the metals. Furthermore, welding takes place in a controlled atmosphere in environments saturated with argon and with the possibility of suctioning the gases produced by the welding itself. In some IR welders, the whole process is performed under a vacuum to further improve the quality of the joints. By uniformly and selectively heating only the area to be welded, the IR emitter allows the user to join metals through brazing, which is performed quickly and very precisely. This prevents the parts from overheating and reduces energy waste [53]. The melting temperature is reached within a short time because the emitter is calibrated to emit radiation with the maximum possible intensity in the shortest possible time. This reduces the duration of the work cycle. The time required to reach the melting temperature depends on the metal being welded. Once the melting temperature is reached, the two pieces to be welded are pressed against each other for the time necessary to ensure perfect welding. The work cycle ends with cooling, followed by the release of the welded component.

Infrared welding can be used in various sectors of dental technology. Given its extreme cleanliness, this welding technique is particularly suitable for metalworking where precision and aesthetic value are important. Moreover, this welding method has a fairly fast learning curve and quickly leads to good results. This is because the method involves reduced combustion and oxidation of the parts to be joined during all the heating phases [54,55]. This welding process is also quite quick and effective when compared to other tools such as a torch [56,57,58].

There are many metals that can be welded using this method, and they also change according to their melting range. For example, base and gold alloys for resins, whose melting range is quite low at 500–700 °C, can be welded. One can then proceed to metal–ceramic alloys with a melting range between 750 and 830 °C or ceramic gold alloys with a melting range between 1000 and 1150 °C. Finally, this type of welding is also applicable to steel and NiCr alloys with a melting range between 1000 and 1250 °C [59].

## 7. Laser Welding

**Figure 5:**
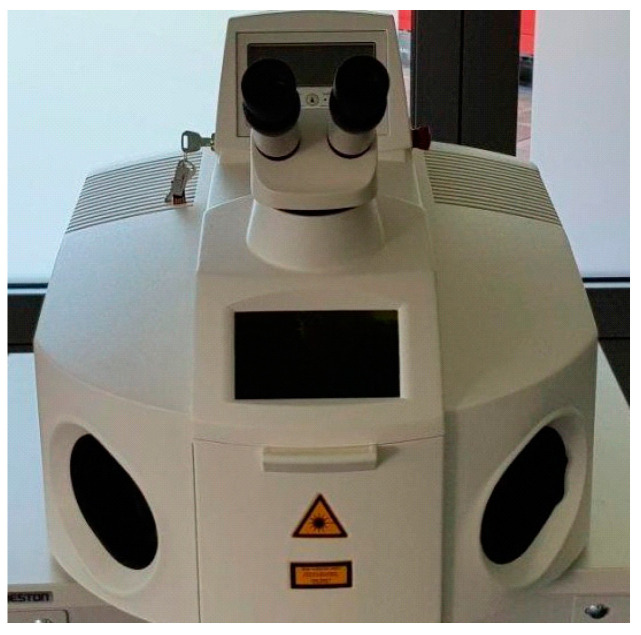
Laser welder.

Laser welding produces metal fusion through a coherent light beam that is focused on the surfaces to be joined (Figure 5). Shielding gas is used to protect the melt, and welding can take place with or without a filler metal. This type of welding allows for high advancement speeds, reduced thermally altered areas, small deformations of the welded piece, the absence of slag and spatter and the possibility of operating through any transparent medium [60,61].

The beam does not need to contact the piece and can be directed, inclined and focused by suitable optical systems. Furthermore, the possibility of transmission through optical fibers makes it particularly suitable for drives, including robotic ones, and for welding complex structures [62,63].

The advantages of laser welding in dental technology compared to traditional braze welding are considerable and make the intervention less invasive and more effective, guaranteeing greater precision and facilitating welding in the vicinity of resins or ceramics without causing damage. Moreover, they allow the user to reduce the amount of heat charged to the areas to be welded, also reducing tensions [8,64,65]. This is most useful when it comes to intervening with a repair; for example, on a damaged prosthesis with a metal framework [66]. Furthermore, this technique allows the user to avoid filler metals, creating mechanically resistant joints with most of the metals used in the dental field. Another advantage is the reduced processing time. This increases the speed of delivery of the finished products to patients and is characterized by a greater precision and resistance to corrosion [67].

In daily practice it is possible to intervene on fixed prostheses such as bridges, copings and implants. In fact, in the latter case, the laser method also allows for titanium-based alloys to be easily welded, with predictable and long-lasting results [68]. The only significant drawback is that the costs of the equipment are still quite high.

## 8. Electric Spot Welding

**Figure 6:**
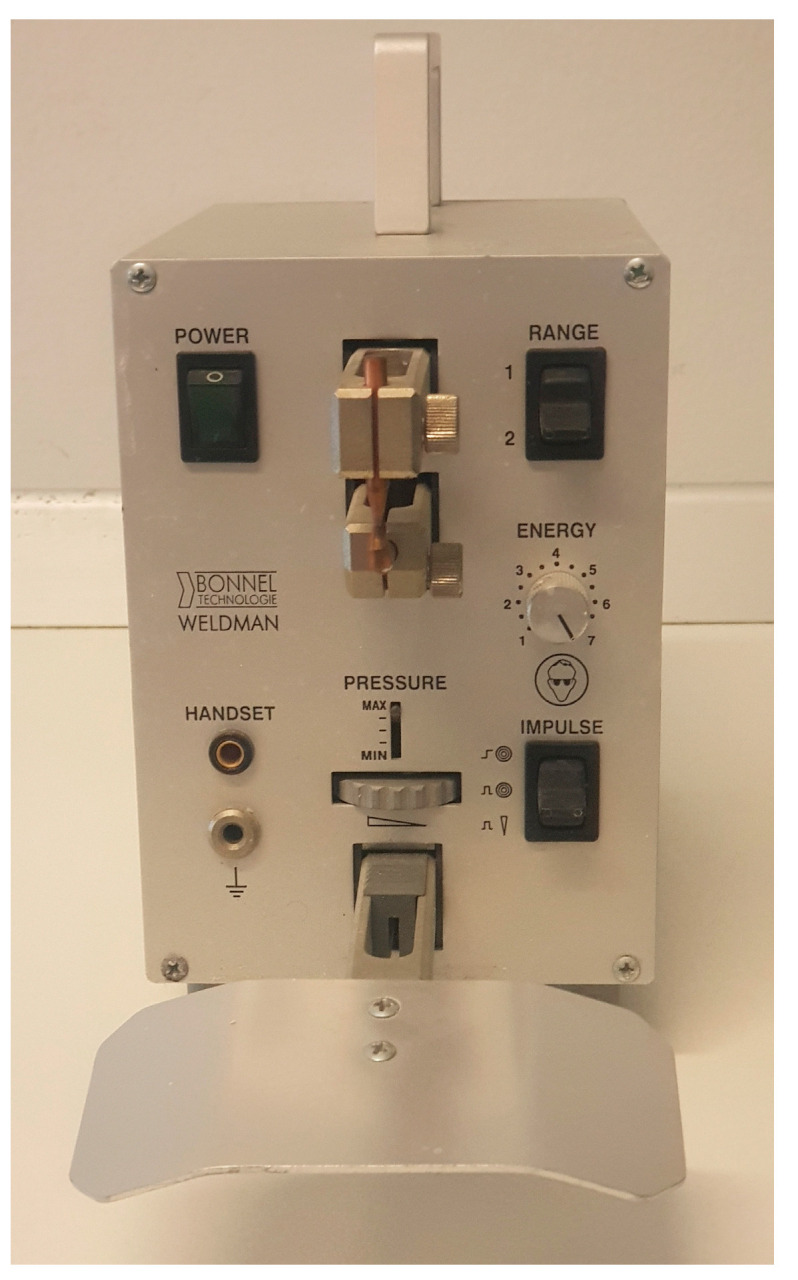
Electrode spotting.

These welding procedures are characterized by the heat generated due to the electrical resistance that opposes the passage of current between two surfaces placed in contact. Furthermore, a certain pressure is applied to the surfaces before, during and after the passage of the electric current in order to improve the final joint (Figure 6). This welding takes place via localized fusion of the base metal without a filler material.

The most important of these is spot welding, which is very common for thin metals such as those used in the construction of orthodontic bands [69]. These welds are also suitable for factories where automation of welding processes is used.

The pieces are brought close to each other in the position in which they should be welded. Two copper or copper alloy electrodes are pressed against the parts to be joined using pliers. For a fraction of a second, a high-intensity current is passed, which develops heat within the area in contact with the electrodes, allowing for localized fusion. The solidification of the metal, which occurs while the electrodes are still pressed, leads to the formation of the welding point [70]. The melting of the metal occurs only within the contact area with the electrodes. This is because, in this area, the electrical resistance is greater, and therefore, the heat generated through the Joule effect is high. This relatively expensive method creates a metal joint that has a poor tensile and fatigue strength [69].

## 9. Intraoral Welding

**Figure 7:**
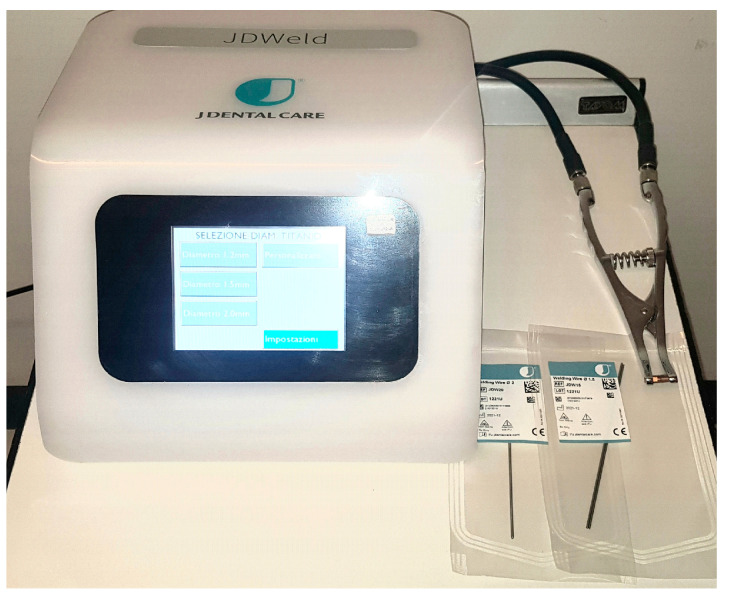
Intraoral welder.

Ultimately, the use of intraoral welding devices in implantology provides benefits to patients such as a reduction in costs and treatment times, with a significant improvement in their quality of life, especially in cases in which the bone crests are particularly affected by atrophy (Figure 7) [71,72].

The technique of intra-oral electric resistance welding enables the fabrication of immediate temporary prostheses on dental implants. This welding method allows for multiple implants to be joined together after their insertion using a titanium bar which is modeled primarily on the patient’s plaster models and then readjusted directly into the oral cavity, reducing any pre-welding tensions [73]. This technique improves the primary stability of the implants, allowing for faster prosthetic rehabilitation. In fact, temporarily fixed prostheses can be applied to the patient on the same day as their surgery. The welding of the titanium implants, according to Mondani, must be performed immediately after the positioning of the implants themselves [74,75]. This allows for a temporary rehabilitation of masticatory function and extremely rapid aesthetics, even in patients subject to severe bone atrophy [76,77]. The provisional prostheses made using this method will later be replaced by definitive prostheses, even if the bar can be kept even in the definitive prosthesis phase. The intraoral welding also reduces one of the causes of implant procedure failure: any movement of the intraosseous implants during the delicate phases of bone healing. In particular, if the bone is particularly atrophic, attention must be paid to the primary stabilization of the implants. This can be improved thanks to the intraoral welding of titanium bars which connect the implants to each other, limiting even micro-movements as much as possible. As this is an intraoral procedure, the level of precision must be remarkable. This technique reduces the errors that could occur in welding carried out only on plaster models, where various inaccuracies derive both from the impression technique and from the realization of the plaster models [78].

The intraoral welders, thanks to a current accumulator and via a delivery clamp, are able to emit a particularly intense electric discharge for an extremely short period (few milliseconds) so that the heat generated does not spread beyond the areas adjacent to the point application of the electrodes. In order to proceed safely, all welding must be carried out while cooling with water. Particular attention must be paid to the fact that the pieces to be joined must be in contact with each other to allow for the passage of current without significant electrical resistance. Intraoral welding also allows dentists to connect already osteointegrated implants to obtain a better connection structure between implants or offer anchorage for removable prosthesis. In some particular clinical conditions, it is also possible to join prosthetic natural teeth and implant screws through the use of appropriate connectors that are capable of correctly distributing the involved forces.

Another application of intraoral welding is related to the possibility of reconstructing the abutments of implants of an inadequate height or shape, or even to reconstruct fractured implants. The procedure involves two different moments that follow each other rapidly: the emission of a first impulse allows for the spot welding of the metal pieces to be welded, blocking their position before a second impulse carries out the actual welding, which takes place via syncrystallisation. There would also be the option of intraoral welding using a fiber-delivered neodymium-doped yttrium aluminum garnet (Nd:YAG) laser. This method, which requires clinical insights, would allow the prosthesis to be positioned and welded intraorally, with predictable results both in terms of aesthetics and patient comfort [79].

## 10. Common Welding Problems

In dentistry, creating complex metal products may require multiple pieces to be made separately and then welded together. This method is necessary when producing the product in a single piece is not feasible or cost-effective. The final product must be able to withstand any loads applied to it [80]. Selecting this connection method brings about some considerable challenges in both clinical and engineering aspects. These challenges include determining the appropriate size and type of welds, which must consider the mechanical stress that the welded pieces will endure during clinical usage [81].

In the welded structure, the lines of tension follow their natural path and tend to accumulate in the geometric discontinuities. As a result, there is an increase in the local stress level and a decrease in the fatigue strength of the parts affected by these joints [82]. In general, all welds are affected by residual stresses. Given the complexity of the thermal cycle with temperatures that vary over time and from point to point, at the end of the welding execution, the joint presents a state of residual stress with an intensity depending on the geometric configuration, degree of constraint and thickness [83]. During welding, the material is subjected to extreme thermal cycling that takes it from room temperature to the melting temperature and then back to room temperature within a short period. This process causes an increase in the volume and changes the shape of the material due to the different distribution of heat. When the material cools, it is unable to return to its original shape due to the presence of the weld and the external constraints imposed on the structure. Residual stresses are also caused by constraints placed on the structure to avoid excessive deformation during welding. This can cause problems, because too stiff constraints tend to reduce deformation while increasing residual stresses [84].

The elastic deformations disappear when the structure returns to room temperature, while the plastic ones are those that generate residual stresses in the structure. The field of residual stresses extends along the length of the joint and depends on the geometry of the joint and on the welding procedure [85]. However, all welded joints are characterized by metallurgical inhomogeneities; that is, different structures in the molten area, in the heat-affected area and in the base material. In some cases, the inhomogeneities lead to significant variations in the mechanical characteristics which must be correctly evaluated during the sizing phase.

While we recognize the merits of soldering, ultimately, a soldered joint represents a potential point of weakness in any dental product. Furthermore, imperfections and defects can also be highlighted within these welded joints. The imperfections represent a discrepancy with respect to the ideal weld. Instead, the defects are to be considered unacceptable imperfections, as they can compromise the resistance of the welded joint.

Weld defects can be divided into five groups: (1) cracks, (2) cavities, (3) solid inclusions, (4) lack of fusion and penetration and (5) defects in shape and size. *Cracks* are the most serious defect because, depending on the size and stresses to which the joint will be subjected, they can be the cause of future breakage. In fact, they represent a discontinuity within the metallic material and, depending on the cause that generates them, they are divided into hot or cold cracks. Hot cracks originate from a high presence of impurities contained in the base metal [86]. The probability of having cracks in the context of a weld increases as the amount of the base metal melted increases. Cold cracks, on the other hand, are caused by the presence of hydrogen in the weld pool [87]. To avoid them, it would be advisable to preheat the components to be welded, also avoiding too rapid cooling. It is also useful to minimize the presence of residual humidity on the metal edges to be joined.

*Cavities* are made up of the absence of material. In fact, any gases present in the bath can remain trapped during very rapid cooling. Depending on their size, the cavities are divided into pores or blowholes. A phenomenon known as termites occurs when several cavities join together, giving rise to an elongated shape. To avoid its formation, it is necessary to decrease the welding speed in order to give the gas time to escape from the pool.

*Solid inclusions* are similar to cavities, but in this weld defect, it is foreign substances that remain trapped in the molten metal. For example, in the case of TIG welding, an infusible tungsten electrode can remain trapped during tungsten welding due to a technical error in the use of the torch.

The *lack of penetration and fusion* can be linked to different problems, but these have in common the absence of continuity between the edges to be joined. The result is often unacceptable and is more easily found in the presence of complex geometries. There is also another type of defect that is similar to the lack of fusion, which is defined as gluing. This can be found in the welds of easily oxidizable alloys. In fact, it occurs when a layer of oxide is interposed between the edge and the fused area. It is a typical defect of steel welded using the MAG technique, arc welding with metal under active gas protection, or oxyacetylene welding. This type of defect is not always identifiable through non-destructive testing; therefore, it is necessary to implement preventive measures to prevent it from forming [88,89].

When a weld has an irregular appearance, presenting defects in shape and size, it has often not been done correctly. The most frequent shape defects are excess stock, when the weld bead is very high. It could be erroneously believed that a thicker weld corresponds to a greater resistance, but on the contrary, due to its shape, tensions are concentrated at the edges of the stock. Under certain clinical conditions, this can drastically reduce the resistance of the joint. It is also possible to have an incomplete filling, which is exactly the opposite of the excess stock. At the basis of this defect, there is excessive penetration or insufficient supply of the material.

## 11. Conclusions

It is clear that in some fields of dentistry, it is thanks to welding that it is possible to obtain the results we see today on our patients both in the field of dental prostheses and in orthodontics. On the other hand, while acknowledging the undeniable advantages of joining via welding, it must be acknowledged that in order to obtain predictable results, the operator must take many factors into consideration. These range from the design to the choice of materials, and from the welding technique to the technical specifications that are adopted. Added to this is the fact that not all welding defects are detectable by eye or through non-destructive testing.

In conclusion, all welding techniques are valid and predictable, but some require a longer learning curve and superior manual skills than others. In any case, it is essential to always treat each operating phase with extreme attention and knowledge.

## Figures and Tables

**Table 1:** Different types of joints.

	Soldering	Brazing	Welding
Temperatures	450 °C	600 °C	3800 °C
	Fusion of filler material only	Heating below the melting point of the pieces to be joined	Heating above the melting point of the pieces to be joined
Filler metal	Yes	Yes	Often used
Mechanical resistance	Poor [no changes]	Good [negligible changes]	Optimal [usually improved]
Field of application	Electronic devices	When the joint does not provide a space between the pieces to be joined	Every field where high mechanical resistance is required
Post-welding heat treatments	Not necessary	Not necessary	Yes
Preheating	Required	Required	Sometimes required depending on the technique used
Skill	Low	Medium	High
Cost	Medium	Medium	High

## Data Availability

The data will be made readily available at a reasonable request from the first author.
